# TIMP-3 Expression Associates with Malignant Behaviors and Predicts Favorable Survival in HCC

**DOI:** 10.1371/journal.pone.0106161

**Published:** 2014-08-29

**Authors:** Xuefeng Gu, Maoying Fu, Yuqin Ding, Huihui Ni, Wei Zhang, Yanfang Zhu, Xiaojun Tang, Lin Xiong, Jiang Li, Liang Qiu, Jiaren Xu, Jin Zhu

**Affiliations:** 1 Department of Infectious Diseases, The First People's Hospital of Kunshan Affiliated with Jiangsu University, Suzhou, China; 2 The Key Laboratory of Cancer Biomarkers, Prevention & Treatment Cancer Center and The Key Laboratory of Antibody Technique of Ministry of Health, Nanjing Medical University, Nanjing, China; 3 Department of Pathology, The Second Affiliated Hospital of Nanjing Medical University, Nanjing, China; 4 Department of Pathology, Jiangsu Province Geriatric Institute, Nanjing, China; 5 Department of Hematology and Oncology, Jiangsu Provincial Hospital, Nanjing, China; 6 Huadong Medical Institute of Biotechniques, Nanjing, China; University of North Carolina School of Medicine, United States of America

## Abstract

The tissue inhibitors of metalloproteinases (TIMPs) are proteins that specifically inhibit the proteolytic activity of the matrix metalloproteinases (MMPs). TIMP-3, the only member of the TIMPs that can tightly bind to the extracellular matrix, has been identified as a unique tumor suppressor that demonstrates the ability to inhibit tumor angiogenesis, invasion, and metastasis. This study aimed to detect the expression of TIMP-3 in hepatocellular carcinoma (HCC) and investigate the association between TIMP-3 expression and its clinicopathological significance in HCC patients. In the current study, reverse transcription-polymerase chain reaction (RT-PCR) and Western blotting of HCC cell lines and one-step quantitative reverse transcription PCR (qPCR) and immunohistochemistry (IHC) analyses in HCC tissues were performed, to characterize the TIMP-3 expression. Kaplan-Meier survival and Cox regression analyses were utilized to evaluate the prognosis of 101 HCC patients. The results showed that the expression of TIMP-3 in HCC was significantly decreased relative to that of non-cancerous cells and tissues. Furthermore, the TIMP-3 expression was statistically associated with malignant behaviors of HCC, including portal vein invasion (p = 0.036) and lymph node metastasis (p = 0.030). Cox regression analysis revealed that TIMP-3 expression was an independent prognostic factor for disease-free survival (p = 0.039) and overall survival (p = 0.049). These data indicate that TIMP-3 expression is a valuable prognostic biomarker for HCC and that TIMP-3 expression suggests a favorable prognosis for HCC patients.

## Introduction

Hepatocellular carcinoma (HCC), the most common primary malignancy of the liver, represents one of the leading causes of cancer mortality worldwide, with over 20,000 deaths in the United States in 2013 [1]. Most HCC cases develop in East and Southeast Asia, however, as China alone accounts for more than 50% of newly diagnosed cases globally (approximately 400,000 cases) [2], [3] and the township of Qidong in the Jiangsu Province in China is one of the highest endemic regions for HCC in the entire world [4]. HCC tumorigenesis is a multistep process, and various factors are associated with HCC development, including hepatitis B (HBV) and hepatitis C (HCV) viral infections, chronic alcohol consumption and nonalcoholic fatty liver disease [5], [6]. Despite various therapeutic strategies for HCC treatment that have improved in the last two decades, such as surgical resection, radiofrequency, microwave ablation, chemotherapy, and transplantation, HCC remains a highly fatal disease because of the high recurrence and metastasis rates [7]; the overall 5-year survival rate of HCC patients has recently been reported to be only 16% [8]. Given the poor prognosis for patients with HCC and the complexity of outcome prediction, it is vital to identify useful prognostic factors for HCC in order to optimize the therapeutic approach for each case.

The tissue inhibitors of metalloproteinases (TIMPs) are proteins that specifically inhibit the proteolytic activity of matrix metalloproteinases (MMPs), and they have been generally recognized as potential suppressors of angiogenesis and tumorigenesis [9], [10]. Among the TIMPs, TIMP-3 has been identified as a unique tumor suppressor and is the only member of the TIMPs that could tightly bind to the extracellular matrix. TIMP-3 has been demonstrated to inhibit tumor angiogenesis, invasion, and metastasis [11]–[13]. TIMP-3 promotes apoptosis in tumor cells through the stabilization of cell surface death receptors and the activation of caspase-8 [14]. Additionally, a large number of clinical studies have evaluated TIMP-3 expression and its clinical significance in a variety of malignant tumors [15]–[17]. Reduced TIMP-3 expression was associated with poor outcomes in esophageal adenocarcinoma and lung cancer patients [18]–[20]. The above data suggest that TIMP-3 operates as a tumor suppressor and that the inhibition of TIMP-3 expression indicates poor survival in human cancer. However, an early report suggested a positive relationship between TIMP-3 promoter methylation and better survival in lung cancer patients [21], and high TIMP-3 expression has been linked to an unfavorable prognosis in head and neck cancer [22]. Hence, the prognostic value of TIMP-3 in human cancers, including HCC, needs to be further elucidated.

In this study, we detected TIMP-3 expression in HCC cell lines via reverse transcription-polymerase chain reaction (RT-PCR) and Western blotting analyses. Furthermore, we examined the TIMP-3 expression in HCC tissues with one-step quantitative-polymerase chain reaction (qPCR) and immunohistochemistry (IHC) analysis of a tissue microarray (TMA). Finally, we evaluated the correlation of TIMP-3 expression with the clinicopathologic features and prognostic significance in HCC.

## Materials and Methods

### Ethics statement

The Ethics Committee of Nanjing Medical University and each local hospital approved the study protocol. Written informed consent was acquired from all of the patients who were enrolled in this study.

### Cell lines

Four HCC cell lines (BEL-7402, SMMC-7721, HepG2 and SK-HEP-1), and one human liver cell line (LO-2) were purchased from the cell bank of the Chinese Academy of Science (Shanghai, China). All cells were cultured in DMEM medium (Gibco, Invitrogen, Carlsbad, CA, USA) supplemented with 10% fetal bovine serum (FBS; Gibco), penicillin (100 U/mL) and streptomycin (100 µg/mL).

### Patient tissue samples

A total of 20 fresh HCC tissues and corresponding adjacent non-cancerous tissues were obtained for this study from the Affiliated Hospital of Nantong University and the First People's Hospital of Kunshan, affiliated with Jiangsu University. Archival tissue samples (101 formalin-fixed, paraffin-embedded HCC tissues and 100 matched tumor-adjacent normal tissues) were obtained from the Affiliated Hospital of Nantong University and the First People's Hospital of Kunshan, affiliated with Jiangsu University, dating from 2003 to 2010. Before surgical therapy, none of the patients had received neoadjuvant chemotherapy, radiation therapy or immunotherapy. Representative and important clinical data, such as gender, age, tumor size, hepatitis B virus (HBV) infection, liver cirrhosis, pathological grade, portal vein invasion, lymph node metastasis, distant metastasis and TNM stage, were collected for further analyses. The TNM stage of all HCC samples was confirmed according to the 2002 American Joint Committee on Cancer/International Union Against Cancer TNM staging system [23].

### RT-PCR and Western blotting analysis in HCC cell lines

For RT-PCR testing, total RNA was extracted from four HCC cell lines (BEL-7402, SMMC-7721, HepG2 and SK-HEP-1) and one human liver cell line (LO-2) using the Trizol reagent (Life Technologies, Inc., Grand Island, NY, USA) according to the manufacturer's guidelines. The prepared RNA (5 µg) was mixed with oligo-dT primers and reverse-transcribed with MMLV reverse transcriptase (Promega, USA). The primers for TIMP-3 were as follows: forward primer 5′- TCT GCA ACT CCG ACA TCG T-3′; reverse primer 5′- TTG GTG AAG CCT CGG TAC AT-3′. β-actin was used as a loading control, and the primers for β-actin were as follows: forward 5′- CTC CAT CCT GGC CTC GCT GT-3′, reverse 5′- GCT GCT ACC TTC ACC GTT CC-3′. PCR amplification was executed in 20 µL using a thermocycler (Biometra, Germany). Total RNA extraction, amplification conditions and RT-PCR procedures were described in our previous publication [24], [25].

For Western blotting analysis, the cells were washed and lysed with cell lysis buffer. Equal amounts of proteins were separated by 10% SDS-PAGE and transferred onto nitrocellulose membranes. The membranes were first incubated with primary anti-TIMP-3 antibody (Abcam, Cambridge, MA, USA), then a secondary antibody, and finally detected with an ECL kit and autoradiography using X-ray film. β-actin blotting was used as an internal control.

### One-step qPCR test and IHC analysis in HCC tissues

For qPCR analysis, 20 fresh HCC tissues and corresponding adjacent non-cancerous tissues were collected. Total RNA was extracted from HCC tissues and non-cancerous tissues following the protocols mentioned above. The glyceraldehyde 3-phosphate dehydrogenase (GAPDH) mRNA level was used to standardize the measurements of the target gene and, with the primers for GAPDH as follows: forward primer 5′-TGC ACC ACC AAC TGC TTA GC-3′; reverse primer 3′-GGC ATG GAC TGT GGT CAT GAG-5′. A SensiMixTM One-Step Kit (Quantace, Berlin, Germany) was used to execute qPCR analysis with a real time PCR system (Bio-Rad Laboratories, Hercules, CA, USA). The one-step qPCR procedure was described in our previous publication [26].

For IHC analysis, 101 HCC tissues and matched non-cancerous tissues were prepared and arranged in a TMA by Alenabio Biotech Co., Ltd (Xi'an, China). The TMA was cut into 4 µm-thick sections and placed on Superfrost charged glass microscope slides. IHC analysis was performed as described previously [24]–[26]. TMA sections were incubated with a primary anti-TIMP-3 antibody (Abcam) in phosphate-buffered saline (PBS), washed and then incubated with a horseradish peroxidase-conjugated antibody (Santa Cruz Biotechnology, Santa Cruz, CA, USA). Negative controls were included by replacement of the primary antibody with PBS. TIMP-3 immunostaining was defined according to the intensity and percentage of TIMP-3-positive tumor cells. The staining intensity was scored as follows: 0 (negative), 1 (weakly positive), 2 (moderately positive), and 3 (strongly positive). The percentage of TIMP-3-positive cells was also classified into 4 categories, in which a score of 1 was given for 0–10%, 2 for 11–50%, 3 for 51–80%, and 4 for 81–100%. The product of the intensity and percentage scores led to the ultimate staining score. The degree of TIMP-3 staining was quantified using a two-level grading system as follows: <3 indicates negative expression while 3–9 indicates positive expression.

### Statistical analysis

The TIMP-3 mRNA expression in fresh HCC tissues relative to the matched non-cancerous tissues was analyzed with the Wilcoxon signed rank nonparametric test. The significance of TIMP-3 protein expression in clinical data from HCC patients was calculated by the chi-square test. Both univariate and multivariate analyses were performed with Cox proportional hazards regression models to identify important factors that were associated with disease-free and overall survival status. The Kaplan-Meier method was utilized to analyze the relationship between TIMP-3 expression and the outcome of HCC patients. The significance level for statistical analysis was set at p<0.05. All statistical analyses were conducted by using SPSS 16.0 (SPSS Inc, Chicago, IL, USA).

## Results

### Summarization of the clinical information from 101 HCC patients

A patient sample of 84 males and 17 females, with a median age of 54.23 years (range 27–73 years), was enrolled in this study. The tumor diameter of 59 patients was >5 cm, while that of the remaining of 42 patients was ≤5 cm. Sixty-seven patients experienced HBV infection and 43 patients encountered liver cirrhosis in their medical history. In terms of the pathological grade of disease, 11 patients were at grade 1, 72 were at grade 2, and 18 were at grade 3. Portal vein invasion was witnessed in 39 patients, lymph node metastasis was detected in 27 patients, and distant metastasis was observed in 7 patients. With regard to the TNM stage among all 101 HCC cases, 13 patients were in stage I, 37 patients were in stage II, 45 patients were in stage III, and the last 6 patients were in stage IV.

### Detection of TIMP-3 expression in HCC cell lines by RT-PCR and Western blotting analysis

**Figure 1:**
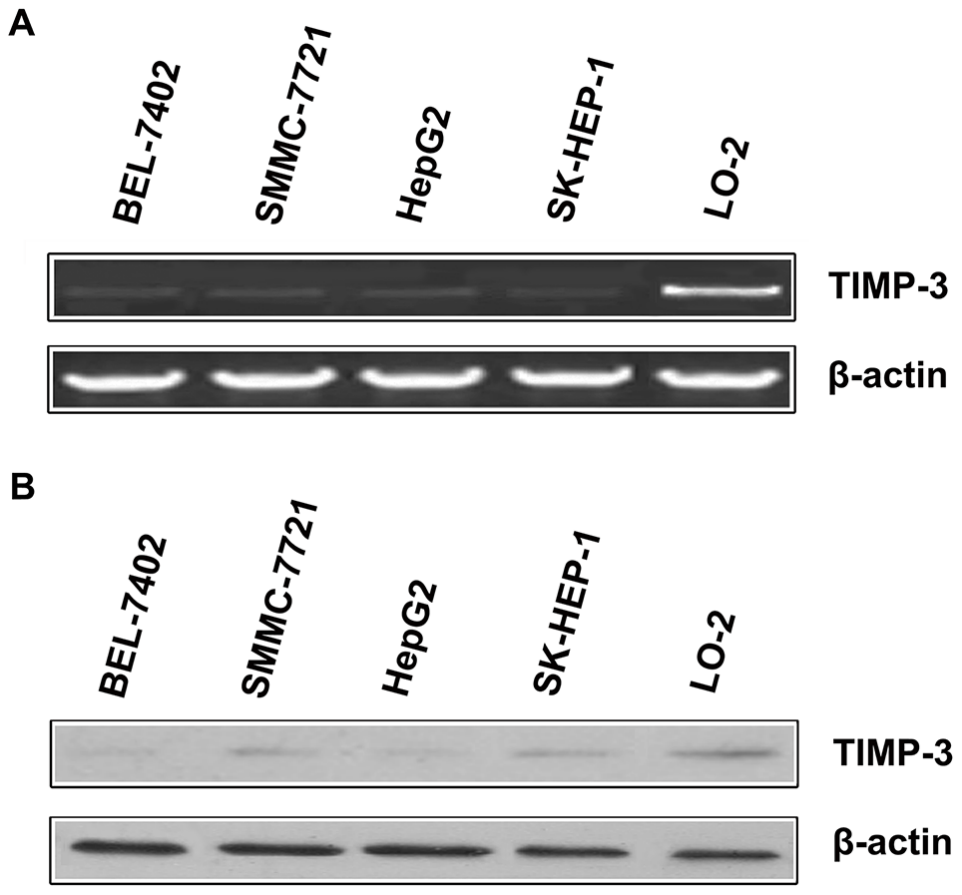
TIMP-3 expression in four hepatocellular carcinoma (HCC) cell lines and one non-cancerous cell line. A. Reverse transcription-polymerase chain reaction (RT-PCR) revealed that TIMP-3 mRNA expression in HCC cell lines was detected at a low intensity when compared to TIMP-3 expression in the human liver cell line, LO-2. B. Western blotting illustrated that TIMP-3 protein expression in the HCC cell lines BEL-7402, SMMC-7721, HepG2 and SK-HEP-1 are decreased relative to the non-cancerous human liver cell line LO-2.

TIMP-3 expression was detected in four HCC cell lines (BEL-7402, SMMC-7721, HepG2 and SK-HEP-1) and one non-cancerous cell line (LO-2) by performing RT-PCR and Western blotting analyses. As shown in Figure 1, RT-PCR and Western blotting analysis revealed that TIMP-3 expression was decreased in four HCC cell lines relative to that of the non-cancerous cell line.

### Detection of TIMP-3 expression in HCC tissues by qPCR

**Figure 2:**
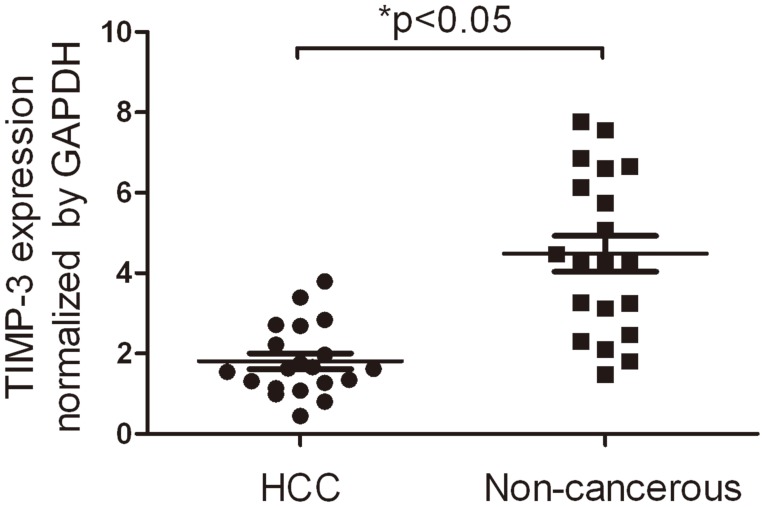
TIMP-3 expression in hepatocellular carcinoma (HCC) tissues and tumor-adjacent, non-cancerous tissues. One-step quantitative real-time polymerase chain reaction (qPCR) demonstrated that the mRNA expression of TIMP-3 in HCC tissues (1.81±0.197) was significantly lower than in matched non-cancerous tissues (4.49±0.446) after normalization with the GAPDH internal control. *p<0.05.

TIMP-3 expression was examined in 20 fresh HCC tissues and their corresponding non-cancerous tissues by qPCR. As shown in Figure 2, the TIMP-3 expression in HCC tissues (1.81±0.197) was significantly lower than that of the corresponding non-cancerous tissues (4.49±0.446) when normalized to GAPDH (p<0.05).

### IHC Detection of TIMP-3 expression in an HCC TMA

**Figure 3:**
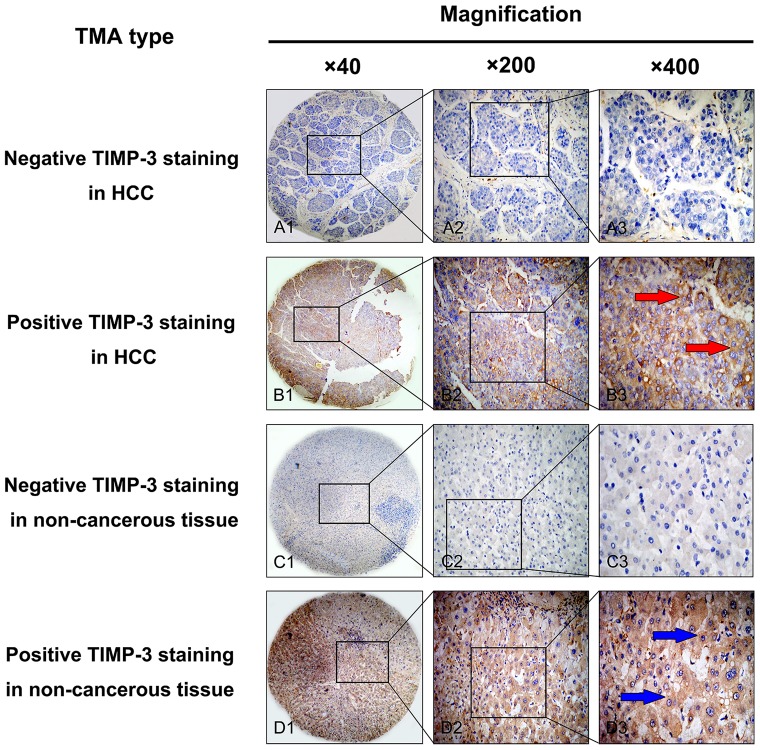
Representative staining pattern of TIMP-3 protein expression in HCC and corresponding non-cancerous tissues in a tissue microarray (TMA). A1, A2 and A3. Negative immunohistochemical (IHC) staining of TIMP-3 in HCC samples. B1, B2 and B3. Positive IHC staining of TIMP-3 in HCC samples. The red arrow highlights positive staining in the cytoplasm of cancer cells. C1, C2 and C3. Negative IHC staining of TIMP-3 in non-cancerous tissue samples. D1, D2 and D3. Positive IHC staining of TIMP-3 in non-cancerous tissue samples. The blue arrow highlights positive staining in the cytoplasm of non-cancerous cells. Original magnification ×40 in A1, B1, C1 and D1; ×200 in A2, B2, C2 and D2; ×400 in A3, B3, C3 and D3.

TIMP-3 expression in HCC tissues was evaluated by IHC analysis. High TIMP-3 expression was exhibited in only 36 of 101 (35.6%) HCC tissue samples, whereas 64 of 101 cases of non-cancerous normal tissues (63.4%) showed positive TIMP-3 expression. The TIMP-3 protein expression level was significantly decreased in HCC tissues when compared to that of non-cancerous tissues (p<0.05). Positive staining was mainly localized in the cytoplasm of HCC cells; representative IHC staining for TIMP-3 expression in HCC is shown in Figure 3. The relationship between TIMP-3 protein expression and clinicopathological characteristics are illustrated in Table 1. It is of note that positive TIMP-3 expression was more prevalent in patients with a lack of portal vein invasion and no lymph node metastasis. Additionally, the statistical results revealed that positive TIMP-3 expression was negatively correlated with portal vein invasion (p = 0.036) and lymph node metastasis (p = 0.030).

**Table 1:** Relationship of high TIMP-3 expression with clinicopathological characteristics in HCC.

Groups	No.	TIMP-3		χ^2^	p value
		+	%		
Total	101	36	35.6		
Gender					
Male	84	28	33.3	1.16	0.281
Female	17	8	47.1		
Age (years)					
<60	77	26	33.8	0.50	0.480
≥60	24	10	41.7		
Tumor size (cm)					
>5	59	20	33.9	0.19	0.664
≤5	42	16	38.1		
Hepatitis B virus infection					
Positive	67	22	32.8	0.68	0.408
Negative	34	14	41.2		
Liver cirrhosis					
Positive	43	16	37.2	0.08	0.777
Negative	58	20	34.5		
Pathological grade					
Grade 1	11	3	27.3	2.42	0.298
Grade 2	72	29	40.3		
Grade 3	18	4	22.2		
Portal vein invasion					
Positive	39	9	23.1	4.37	0.036*
Negative	62	27	43.5		
Lymph node metastasis					
Positive	27	5	18.5	4.71	0.030*
Negative	74	31	41.9		
Distant metastasis					
Positive	7	1	14.3	1.50	0.221
Negative	94	35	37.2		
TNM stage					
Stage I	13	4	30.8	5.27	0.153
Stage II	37	17	45.9		
Stage III	45	15	33.3		
Stage IV	6	0	0.0		

*p<0.05.

### Survival analysis

**Figure 4:**
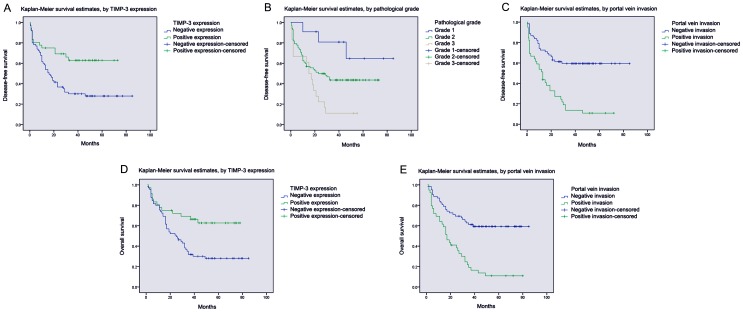
Survival analysis of HCC patients by the Kaplan-Meier method. A. The disease-free survival rate in patients with negative TIMP-3 expression (blue line) was significantly lower than that of patients with positive TIMP-3 expression (green line). B. The disease-free survival rate in patients with pathological grade 1 (blue line) and 2 (green line) was significantly higher than that of patients with pathological grade 3 (yellow line). C. The disease-free survival rate in patients with positive portal vein invasion (green line) was significantly lower than that of patients without portal vein invasion (blue line). D. The overall survival rate in patients with negative TIMP-3 expression (blue line) was significantly lower than that in patients with positive TIMP-3 expression (green line). D The overall survival rate in patients with positive portal vein invasion (green line) was significantly lower than that of patients without portal vein invasion (blue line).

According to univariate analysis, several items were correlated with both disease-free survival and overall survival of HCC, including TIMP-3 expression, tumor size, portal vein invasion, lymph node metastasis and the TNM stage. In addition, distant metastasis also affected disease-free survival, but not overall survival (Tables 2 and 3). Moreover, by using multivariate analysis with Cox regression model, we found that TIMP-3 expression (p = 0.039, p = 0.049) and portal vein invasion (p = 0.030, p = 0.019) were associated with disease-free survival and overall survival, respectively. Additionally, portal vein invasion (p = 0.044) was also correlated with disease-free survival of HCC (Tables 2 and 3). Kaplan-Meier survival curves subsequently indicated that HCC patients with positive TIMP-3 expression and negative portal vein invasion displayed a statistically better duration of disease-free survival and overall survival (Figure 4). Meanwhile, HCC patients with an advanced pathological grade encountered unfavorable disease-free survival time (Figure 4).

**Table 2:** Univariate and multivariate analysis of prognostic factors in HCC for disease-free survival.

	Univariate analysis			Multivariate analysis		
	HR	p>|z|	95% CI	HR	p>|z|	95% CI
TIMP-3 expression						
High versus Low	0.40	0.004*	0.217–0.749	0.50	0.039*	0.257–0.967
Gender						
Male versus Female	1.18	0.640	0.582–2.409			
Age (years)						
<60 versus ≥60	1.68	0.137	0.848–3.317			
Tumour size (cm)						
>5 versus ≤5	1.79	0.035*	1.042–3.075	1.18	0.581	0.655–2.128
Hepatitis B virus infection						
Positive versus Negative	1.23	0.465	0.706–2.142			
Liver cirrhosis						
Positive versus Negative	0.90	0.701	0.536–1.523			
Pathological grade						
Grade 1 and 2 versus Grade 3	2.15	0.001*	1.344–3.342	1.67	0.044*	1.015–2.761
Portal vein invasion						
Positive versus Negative	3.21	0.001*	1.909–5.396	1.93	0.030*	1.066–3.510
Lymph node metastasis						
Positive versus Negative	2.64	0.001*	1.543–4.516	1.71	0.086	0.927–3.159
Distant metastasis						
Positive versus Negative	2.34	0.038*	1.049–5.207	0.63	0.334	0.247–1.609
TNM stage						
Stage I–II versus Stage III–IV	0.32	0.001*	0.184–0.564	0.56	0.093	0.282–1.103

*p<0.05.

**Table 3:** Univariate and multivariate analysis of prognostic factors in HCC for overall survival.

	Univariate analysis			Multivariate analysis		
	HR	p>|z|	95% CI	HR	p>|z|	95% CI
TIMP-3 expression						
High versus Low	0.41	0.005*	0.220–0.759	0.52	0.049*	0.267–0.997
Gender						
Male versus Female	1.20	0.512	0.691–2.097			
Age (years)						
<60 versus ≥60	1.73	0.113	0.877–3.428			
Tumour size (cm)						
>5 versus ≤5	1.78	0.036*	1.037–3.062	1.27	0.422	0.709–2.272
Hepatitis B virus infection						
Positive versus Negative	1.20	0.512	0.691–2.097			
Liver cirrhosis						
Positive versus Negative	0.86	0.563	0.508–1.446			
Pathological grade						
Grade 1 and 2 versus Grade 3	2.19	0.001*	1.369–3.511	1.60	0.052	0.996–2.556
Portal vein invasion						
Positive versus Negative	3.27	0.001*	1.939–5.501	2.04	0.019*	1.124–3.712
Lymph node metastasis						
Positive versus Negative	2.42	0.001*	1.420–4.132	1.40	0.285	0.789–2.557
Distant metastasis						
Positive versus Negative	2.07	0.072	0.936–4.594			
TNM stage						
Stage I–II versus Stage III–IV	0.35	0.001*	0.199–0.606	0.66	0.237	0.338–1.307

*p<0.05.

## Discussion

The MMPs, a family of extracellular proteolytic enzymes, and their cognate inhibitors (TIMPs) are both known to play significant roles during tumor development. A proper balance between MMPs and TIMPs seems to be of great importance for influencing cancer metastasis and invasion [9], [27]–[29]. Tumor cells can synthesize MMPs and influence cellular properties, including cell growth, death and migration, as well as contribute to the invasion, angiogenesis, and establishment of metastatic lesions in the tumor environment [28], [30]. TIMPs, which are natural inhibitors of MMPs and contain four members (denoted TIMP-1 to -4), is reported to ameliorate the invasion and metastasis of tumor cells induced by MMPs [20], [31]. As for TIMP-3, a large number of studies have shown that the expression of TIMP-3 was reduced in various cancer tissues when compared to non-cancerous tissues or in the advanced stages of cancer relative to the early stages of cancer. The decreased expression of TIMP-3 was significantly associated with pathologic stage, nodal involvement, and poor survival [16]–[20]. The possible mechanisms of TIMP-3 function are a subject of much investigation. TIMP-3 suppresses tumorigenesis and angiogenesis by interacting with Integrin α7 and angiotensin II type 2 receptor [32], [33]. TIMP-3 induces endothelial apoptosis in lung cancer by inhibiting p-AKT and inducing p-ERK1/2 pathways [13]. The expression of TIMP-3 could be repressed by zeste homolog 2 and result in cancer cell migration [34]. TIMP-3 expression in tumors can also be modulated through the regulation of microRNAs, as TIMP3 is a positive target of several microRNAs including miR21, miR181b, miR221 and 222 [35]–[37]. All data suggest that the inhibitory effect of TIMP-3 in cancer development could identify TIMP-3 as a novel and useful biomarker in human cancer. However, the clinicopathological significance, particularly the prognostic role of TIMP-3 in HCC, has not been investigated. The potential of TIMP-3 as a candidate for targeted therapy in HCC requires further exploration.

The RT-PCR and Western blotting analyses illustrated that TIMP-3 mRNA and protein expression was significantly reduced in four HCC cell lines when compared to that of non-cancerous cell lines. Subsequently, qPCR further illustrated that the TIMP-3 mRNA expression levels in HCC tissues were lower than those in normal non-cancerous tissues. Furthermore, IHC analysis of an HCC TMA similarly showed a reduced cytoplasmic expression of TIMP-3 protein in cancer cells relative to normal non-cancerous cells. These results are similar to the studies that indicated that the expression of TIMP-3 was inhibited in malignant cancers [16], [18], [20], [38]. Moreover, positive TIMP-3 expression in HCC was negatively correlated with certain clinical pathologic items, including portal vein invasion and lymph node metastasis. Likewise, Zhang et al. reported that high TIMP-3 expression inhibited tumorigenic and metastatic potential in HCC xenografts [39]. Wang et al. concluded that the upregulation of hepatic miR-181b promoted hepatocarcinogenesis by inhibiting TIMP-3 [36]. Our results are in line with previous studies and further confirm that the positive expression of TIMP-3 may play a critical role in inhibiting malignant behaviors in HCC, including portal vein invasion and lymph node metastasis.

Univariate analysis and multivariate analysis demonstrated that TIMP-3 expression and portal vein invasion were both correlated with life span in the disease-free survival and the overall survival of HCC patients. Moreover, Kaplan-Meier analysis analogously demonstrated that the life span of patients with positive TIMP-3 expression was longer than that of patients with negative expression. These data are also consistent with previous reports that low TIMP-3 expression facilitated tumor development and predicted poor survival in several human cancers [18]–[20]. Conclusively, we believe that TIMP-3 significantly exerts anti-oncogenic roles and that positive TIMP-3 expression critically suspends malignant activities associated with cancer invasion. It is rational to presume that TIMP-3 could be used as a novel candidate for cancer therapy. To date, several therapeutic strategies targeting TIMP-3 have already shown promising effectiveness in cancer treatment [40], [41].

However, some contradictory results imply that TIMP-3 expression is increased in certain types of cancers and that high TIMP-3 expression is linked poor prognosis in head and neck cancer [21], [42], [43]. A possible explanation for the conflicting data may be attributed to differences in tumor origins and antibody quality [42]. Further studies that enroll a larger number of clinical HCC patients are necessary to validate our results that characterize TIMP-3.

In summary, this is the first study to illustrate that positive TIMP-3 expression is correlated with the malignant phenotype of HCC. The data from the current study imply that TIMP-3 could be defined as a novel biomarker for HCC prognosis and that targeting TIMP-3 may provide a promising strategy for HCC treatment.

## Supporting Information

Table S1 **Clinical data of 101 HCC patients for statistical analysis.**
(XLS)
